# RBMS1 Coordinates with the m^6^A Reader YTHDF1 to Promote NSCLC Metastasis through Stimulating S100P Translation

**DOI:** 10.1002/advs.202307122

**Published:** 2024-02-11

**Authors:** Yu Sun, Dan Chen, Siwen Sun, Menglin Ren, Liang Zhou, Chaoqun Chen, Jinyao Zhao, Huanhuan Wei, Qingzhi Zhao, Yangfan Qi, Jinrui Zhang, Ge Zhang, Han Liu, Qingkai Yang, Quentin Liu, Yang Wang, Wenjing Zhang

**Affiliations:** ^1^ Sino‐US Research Center for Cancer Translational Medicine of the Second Affiliated Hospital of Dalian Medical University & Institute of Cancer Stem Cell Dalian Medical University Dalian 116023 China; ^2^ Department of Pathology the First Affiliated Hospital of Dalian Medical University Dalian Medical University Dalian 116011 China; ^3^ Department of Oncology & Sino‐US Research Center for Cancer Translational Medicine the Second Affiliated Hospital Dalian Medical University Dalian 116023 China; ^4^ Institute of Cancer Stem Cell Dalian Medical University Dalian 116044 China; ^5^ CAS Key Laboratory of Computational Biology Bio‐Med Big Data Center Shanghai Institute of Nutrition and Health University of Chinese Academy of Sciences Chinese Academy of Sciences Shanghai 200031 China; ^6^ Department of Immunology College of Basic Medical Sciences Dalian Medical University Dalian 116044 China

**Keywords:** lung cancer, metastasis, RBMS1, RNA binding protein

## Abstract

Metastasis is the leading cause for the high mortality of lung cancer, however, effective anti‐metastatic drugs are still limited. Here it is reported that the RNA‐binding protein RBMS1 is positively associated with increased lymph node metastasis in non‐small cell lung cancer (NSCLC). Depletion of RBMS1 suppresses cancer cell migration and invasion in vitro and inhibits cancer cell metastasis in vivo. Mechanistically, RBMS1 interacts with YTHDF1 to promote the translation of S100P, thereby accelerating NSCLC cell metastasis. The RRM2 motif of RBMS1 and the YTH domain of YTHDF1 are required for the binding of RBMS1 and YTHDF1. RBMS1 ablation inhibits the translation of S100P and suppresses tumor metastasis. Targeting RBMS1 with NTP, a small molecular chemical inhibitor of RBMS1, attenuates tumor metastasis in a mouse lung metastasis model. Correlation studies in lung cancer patients further validate the clinical relevance of the findings. Collectively, the study provides insight into the molecular mechanism by which RBMS1 promotes NSCLC metastasis and offers a therapeutic strategy for metastatic NSCLC.

## Graphical abstract

Metastasis is the leading cause of lung cancer, however, the mechanisms involved in non‐small cell lung cancer (NSCLC) metastasis are not fully clarified and effective anti‐metastatic drugs are still limited. RNA‐binding motif, single‐stranded‐interacting protein 1 (RBMS1) is a promising anti‐metastatic target and the RBMS1 inhibitor nortriptyline hydrochloride (NTP) is a potent therapeutic agent for anti‐metastatic therapy in human lung cancer.

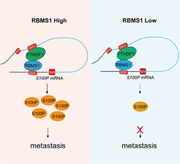

## 1 Introduction

Lung cancer is one of the most commonly diagnosed cancers, which is the leading cause of cancer death in the world.^[^ ^1^, ^2^ ^]^ Non‐small cell lung cancer (NSCLC) accounts for 85% of lung cancer and encompasses multiple cancer types, including adenocarcinomas (LUADs), squamous cell cancers (LUSCs), and large cell cancers.^[^ ^3^ ^]^ Although rapid advances in diagnostic techniques, molecular‐targeted drugs, and immune checkpoint therapy, the five‐year overall survival rate of NSCLC patients remains quite low.^[^ ^4^, ^5^, ^6^, ^7^ ^]^ Metastasis is the leading cause of cancer‐related death.^[^ ^8^ ^]^ Unfortunately, the mechanisms involved in NSCLC metastasis have not been fully clarified yet. Therefore, exploration of the mechanisms underlying NSCLC metastasis will essentially help develop effective metastatic biomarkers and potential therapeutic targets to improve the patient's survival rate.

RNA binding proteins (RBPs) are a diverse class of proteins containing unique RNA‐binding domains. RBPs form various dynamic ribonucleoprotein complexes with RNA molecules to control various aspects of gene expression, including RNA splicing, mRNA stability, mRNA localization, translation, and so on.^[^ ^9^ ^]^ Given that RBPs are key regulators of gene expression, alterations of these proteins are generally implicated in human diseases, including cancer.^[^ ^10^, ^11^ ^]^ In addition, RBPs are largely underestimated contributors in tumorigenesis, which are involved in apoptosis, the epithelial‐mesenchymal transition (EMT), DNA repair, autophagy, cell proliferation, immune response, and metabolism.^[^ ^12^ ^]^ RNA‐binding motif, single‐stranded‐interacting protein 1 (RBMS1), also known as MSSP1, is often overexpressed in malignant cells, including NSCLC.^[^ ^13^, ^14^, ^15^ ^]^ The major biological functions of RBMS1 include regulating DNA replication, DNA transcription, RNA stability, and translation.^[^ ^15^, ^16^, ^17^, ^18^ ^]^ The exact role of RBMS1 in cancer progression remains controversial. RBMS1 can promote gastric cancer metastasis through autocrine IL‐6/JAK2/STAT3 signaling.^[^ ^13^ ^]^ Meanwhile, loss of RBMS1 promotes anti‐tumor immunity through enabling PD‐L1 checkpoint blockade in triple‐negative breast cancer.^[^ ^14^ ^]^ These data suggest that RBMS1 is a pro‐oncogene. Nevertheless, other studies suggested that RBMS1 might be a potential tumor suppressor.^[^ ^18^, ^19^ ^]^ RBMS1 suppresses colon cancer metastasis through targeting stabilization of multiple genes, including the tumor suppressor AKAP12 and a WNT pathway interacting protein, SDCBP.^[^ ^18^ ^]^ In our previous study, we found that RBMS1 is upregulated in lung cancer and depletion of RBMS1 inhibits lung cancer cell growth.^[^ ^15^ ^]^ However, the role of RBMS1 in the regulation of NSCLC metastasis has not been documented yet.

S100P is a member of the S100 calcium‐binding protein family that has been reported to have intracellular and extracellular functions.^[^ ^20^ ^]^ S100P is overexpressed in a variety of cancers, including NSCLC, and its expression is associated with metastasis, drug resistance, and poor clinical outcome.^[^ ^21^, ^22^ ^]^ Meta‐analysis of cDNA array data revealed that S100P was one of five genes dysregulated in lung cancer.^[^ ^23^ ^]^ Moreover, S100P had been demonstrated to be overexpressed in metastatic tissues of NSCLC.^[^ ^24^ ^]^ Moreover, knockdown of S100P inhibited cell migration in highly invasive NSCLC cells.^[^ ^25^ ^]^

Here, our results showed that upregulation of RBMS1 is associated with increased lymph node metastasis in NSCLC. Enhanced expression of RBMS1 promotes NSCLC cell migration and invasion in vitro and in vivo by interacting with YTHDF1 to stimulate the translation of S100P. Our study reveals RBMS1 acts as an important metastatic promoter by modulating S100P translation, suggesting that RBMS1 may be a promising potential target for metastatic NSCLC.

## 2 Results

### 2.1 RBMS1 Is Upregulated in Metastatic NSCLC Specimens

**Figure 1:**
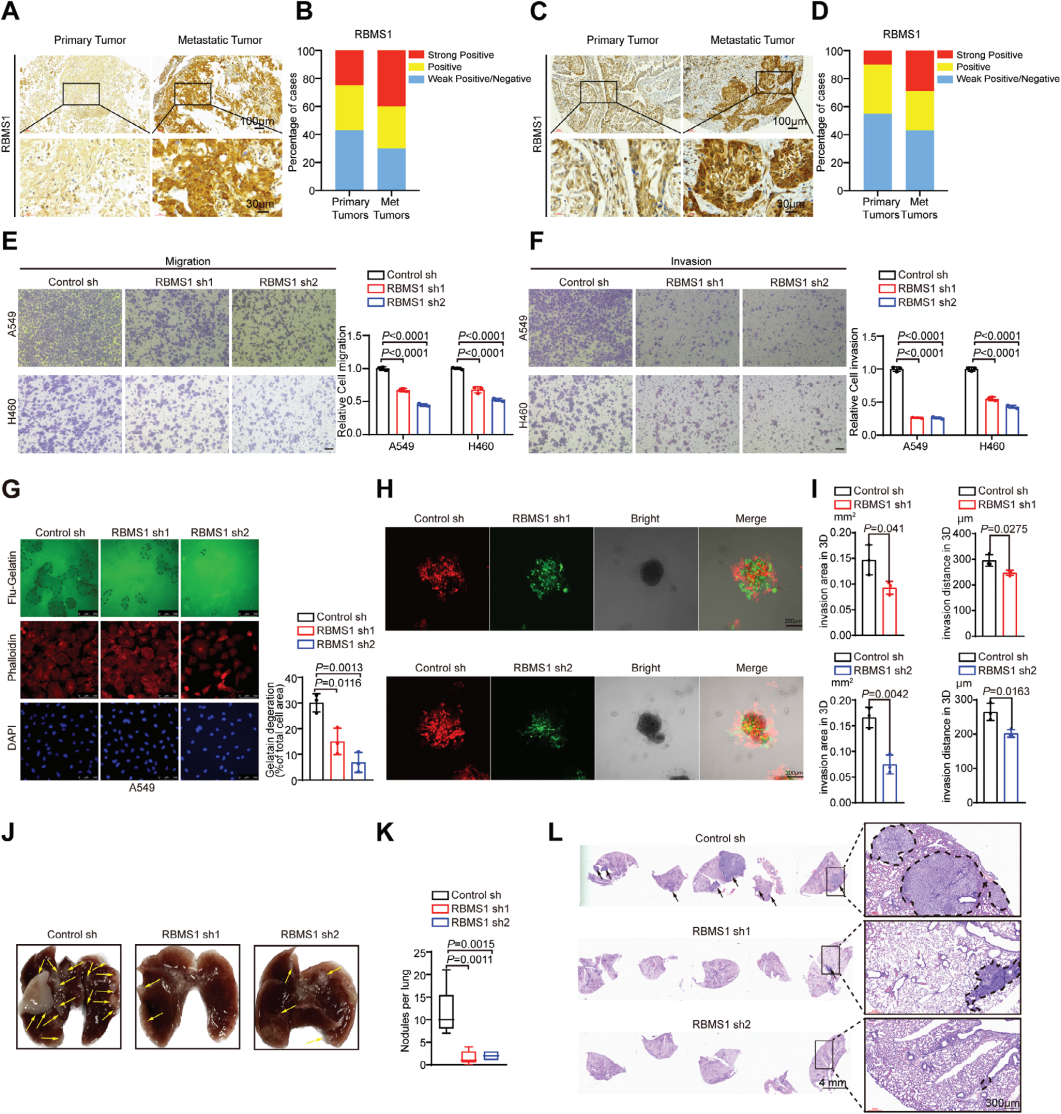
RBMS1 deficiency inhibits cell migration and invasion in vitro and metastasis in vivo. A) Representative images from immunohistochemical staining of RBMS1 in metastatic lymph nodes (*n* = 30) and matched primary lung adenocarcinoma tissues (*n* = 30). Scale bars: 100 µm (top) and 30 µm (bottom). B) The quantification of RBMS1 protein levels in metastatic lymph nodes and primary lung adenocarcinoma tissues. The RBMS1 levels were classified into 3 grades (weak positive/negative, positive, strong positive) based on quantification of immunohistochemical staining and plotted. C) Representative images from immunohistochemical staining of RBMS1 in metastatic lymph nodes (*n* = 30) and matched primary lung squamous cell carcinoma tissues (*n* = 30). Scale bars: 100 µm (top) and 30 µm (bottom). D) The quantification of RBMS1 protein levels in metastatic lymph nodes and primary lung squamous cell carcinoma tissues. The RBMS1 levels were classified into 3 grades (weak positive/negative, positive, strong positive) based on quantification of immunohistochemical staining and plotted. E,F) Effect of RBMS1 knockdown on migration and invasion of A549 and H460 cells evaluated by transwell assays. Scale bars: 100 µm. *P* values were determined using one‐way ANOVA with Dunnett's multiple comparison test (*n* = 3). G) Effect of RBMS1 knockdown on invadopodia function was measured by gelatin degradation assay in A549. 2000 cells were plated onto FITC‐gelatin substrates (Green) and cultured for 36 h. Following staining with Cy3‐phalloidin (Red) and DAPI (Blue), cells were imaged using immunofluorescence microscopy, and representative images are shown. The degraded areas were quantified by Image J software. Scar bars, 100 µm. *P* values from ordinary one‐way ANOVA with Dunnett's multiple comparison test (*n* = 3). H) The 3D matrix multicellular spheroids migration assay was used to examine invasion ability of RBMS1. Immunofluorescence images of the tumor spheroids of mCherry labeled control A549 cells and GFP labeled RBMS1 knockdown A549 cells after 96 h of culture were shown. I) Quantification of the invasion area and distance of A549 control sh cells (Red) and A549 RBMS1 sh1 cells (Green) in 3D matrix multicellular spheroids migration assay. Data are presented as mean ± SD of three independent experiment. *P* values were determined using unpaired *t* test (*n* = 3). J) Representative images of pulmonary tumors formed by injecting control sh, RBMS1 sh1, and RBMS1 sh2 A549 cells into nude mice via tail vein. K) The number of lung metastatic nodules in all five lung lobes from each mouse was counted and statistically analyzed. (*n* = 5 for each group). *P* values were determined using one‐way ANOVA with Dunnett's multiple comparison test. L) The pulmonary metastases in the mouse model were histologically analyzed by H&E staining. Scar bars, 4 mm (left). Representative lung metastatic nodules are shown on right. Scar bars, 300 µm (right).

We have previously reported that RBMS1 is upregulated in lung cancer and high RBMS1 expression is associated with poor prognosis in lung cancer patients.^[^ ^15^ ^]^ To further explore the relationship between RBMS1 expression and clinicopathological features of lung cancer patients, forty tumor tissues were assigned to two groups (high or low RBMS1 expression) based on the immunohistochemistry (IHC) score. Importantly, we found that higher RBMS1 expression level was significantly correlated with clinical stage (*P* = 0.0491) and lymph node metastasis (*P* = 0.028) in lung cancer patients, but not with other factors, including sex, age, and tumor size (**Table** **1**). We further determined RBMS1 expression by IHC using two tissue microarrays consisting of 30 primary NSCLC tissues and matched metastatic tissues, respectively. We revealed that RBMS1 expression level was higher in most metastatic tumors than that in the matched primary tumors (**Figure** **1**A–D). Taken together, our data suggest that high RBMS1 expression is associated with lung cancer metastasis.

**Table 1:** Correlation between RBMS1 expression and clinicopathological characteristics of NSCLC patients.

Characteristics	RBMS1		*P*
	Low no. cases [%]	High no. case [%]	Chi‐squared test *P*‐value
Age (years)			
>65	13 (56.5%)	10 (43.5%)	0.5535
≤65	8 (47.1%)	9 (52.9%)	
Gender			
Male	16 (50.0%)	16 (50.0%)	0.5266
Female	5 (62.5%)	3 (37.5%)	
Clinical stage			
I/II	18 (62.1%)	11 (37.9%)	0.0491^a)^
III	3 (27.3%)	8 (72.7%)	
Tumor size			
≤5 cm	11 (55.0%)	9 (45.0%)	0.7515
>5 cm	10 (50.0%)	10 (50.0%)	
Lymph node metastasis			
Negative	16 (66.7%)	8 (33.3%)	0.028^a)^
Positive	5 (31.2%)	11 (68.8%)	
T States			
T1	6 (66.7%)	3 (33.3%)	0.6024
T2	11 (50.0%)	11 (50.0%)	
T3	4 (44.4%)	5 (55.6%)	

^a)^ *P* < 0.05 was considered significant.

### 2.2 RBMS1 Deficiency Inhibits Cell Migration and Invasion In Vitro and Metastasis In Vivo

To investigate the potential effect of RBMS1 on lung cancer metastasis, we stably depleted RBMS1 in A549, H460 and H2170 lung cancer cells using two short‐hairpin RNAs (shRNAs). Meanwhile, RBMS1 was stably overexpressed in both A549 and H460 lung cancer cells. The depletion and overexpression efficiencies of RBMS1 were confirmed by a western blot assay (Figure S1A,B, Supporting Information). We subsequently performed transwell migration and invasion assays to evaluate the effect of RBMS1 expression on cell migration and invasion of NSCLC cells. As expected, RBMS1 knockdown significantly decreased the migration and invasion abilities of A549, H460 and H2170 cells as compared with control cells (Figure 1E,F and Figure S1C, Supporting Information), whereas RBMS1 overexpression promoted these abilities (Figure S1D,E, Supporting Information). Invadopodia, actin‐rich membrane protrusions, could degrade the surrounding extracellular matrix for invasion. We next examined the effect of RBMS1 knockdown on invadopodia function by gelatin degradation assay in A549 cells. The function of invadopodia was determined by co‐localizing the actin cytoskeleton and nuclei with fluorescent gelation degradation sites. Consistently, RBMS1 knockdown reduced the invasive ability of A549 cells, as indicated by decreased areas devoid of fluorescence where cells have degraded the matrix (Figure 1G). Moreover, a microfluidic 3D coculture model^[^ ^26^ ^]^ was applied to compare the invasion ability of RBMS1 depleted and control A549 cells. GFP‐labeled RBMS1 knockdown A549 cells and mCherry‐labeled control A549 cells were seeded on the concave microdevice in a ratio of 1:1. The seeded microfluidic device was maintained in a humidified atmosphere of 95% air and 5% CO_2_ at 37 °C for 24 h to form cell spheroids. Cell spheroids were subsequently released and cultured for 3 d. The invasive capacity of cells was determined by analyzing invasion area and distance. The decrease in the out‐migration area and the distance of A549 cells with RBMS1 depletion indicates that the invasiveness of A549 cells was significantly inhibited by RBMS1 knockdown (Figure 1H,I).

To confirm these findings, we further examined the effect of RBMS1 knockdown on lung cancer metastasis in vivo. We established a mouse lung cancer metastasis model by injecting the mouse tail vein with A549 cells stably depleted RBMS1 or control. Consistent with the in vitro analyses, RBMS1 knockdown reduced the number of metastatic lung nodules as compared to the control group (Figure 1J,K). H&E staining of excised lung sections confirmed the lower frequency of metastases in RBMS1‐depleted tumors (Figure 1L). Collectively, these results indicate that RBMS1 deficiency inhibits lung cancer metastasis in vitro and in vivo.

### 2.3 Depletion of RBMS1 Inhibits Lung Cancer Metastasis Partially by Reducing S100P

**Figure 2:**
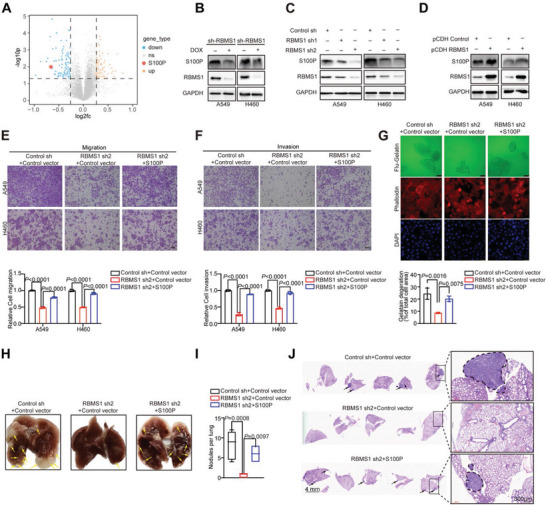
Depletion of RBMS1 inhibits lung cancer metastasis partially by reducing S100P. A) The volcano map showing the differentially expressed proteins from quantitative proteomics using A549 cells with or without depleted RBMS1. B) The protein levels of RBMS1 and S100P were examined in A549 and H460 cells with doxycycline‐induced depletion of RBMS1. C) The protein levels of RBMS1 and S100P were measured in A549 and H460 cells with stable knockdown of RBMS1. D) The protein level of S100P and RBMS1 were examined in A549 and H460 cells with RBMS1 overexpression. E,F) The effect of S100P on migration and invasion in RBMS1 stably depleted A549 and H460 cells were determined by transwell assay. Scale bars: 100 µm. *P* values were determined using one‐way ANOVA with Tukey's multiple comparison test (*n* = 3). G) Effect of S100P on invadopodia function was tested by gelatin degradation assay in RBMS1 stably depleted A549 cells. The degraded areas were quantified by Image J software. Scar bars, 50 µm. *P* values were determined by one‐way ANOVA with Tukey's multiple comparison test (*n* = 3). H) Effect of S100P on tumor metastasis potential in RBMS1‐knockdown A549 cells was examined by tail vein injection metastasis model. After 13 weeks mice were killed, and the representative brightfield lung images of each group are shown. I) The number of lung metastatic nodules in all five lung lobes from each mouse was counted and statistically analyzed. (*n* = 5 for each group). *P* values were determined by one‐way ANOVA with Tukey's multiple comparison test. J) The pulmonary metastases in the mouse model were histologically analyzed by H&E staining. Scar bars, 4 mm (left). Representative lung metastatic nodules are shown. Scar bars, 300 µm (right).

To investigate the underlying molecular mechanisms through which RBMS1 regulates lung cancer metastasis, we identified the differentially expressed proteins based on previously reported quantitative proteomics data in lung cancer cells with doxycycline‐induced depletion of RBMS1.^[^ ^15^ ^]^ Intriguingly, the protein level of S100P, a member of the S100 calcium‐binding protein family, was significantly decreased upon RBMS1 depletion (**Figure** **2**A and Table S1, Supporting Information). Such result was verified in A549 and H460 lung cancer cells with doxycycline‐induced depletion of RBMS1 (Figure 2B). S100P has been reported to be upregulated in multiple cancers and associated with metastasis and poor prognosis.^[^ ^22^ ^]^ Moreover, the level of S100P was further confirmed to be significantly reduced in A549, H460, and H2170 lung cancer cells with stable or transient knockdown of RBMS1 (Figure 2C and Figure S2A,B, Supporting Information). Conversely, overexpression of RBMS1 increased the level of S100P in both A549 and H460 cells (Figure 2D). In addition, overexpression of S100P significantly promoted cancer cell migration and invasion in A549 and H460 cells (Figure S2C–E, Supporting Information). Importantly, restoration of S100P almost fully reversed the RBMS1 depletion‐induced inhibition of cell migration and invasion in A549 and H460, as judged by transwell migration and invasion assays (Figure 2E,F and Figure S2F, Supporting Information). Moreover, gelatin degradation assay further verified that S100P overexpression reversed the RBMS1 knockdown‐induced invasion inhibition (Figure 2G). Consistently, RBMS1 depletion‐induced the reduction in the number of metastatic lung nodules was also significantly rescued with restoration of S100P in a mouse lung metastasis model (Figure 2H–J).

### 2.4 RBMS1 Coordinates with YTHDF1 to Regulate S100P Translation

**Figure 3:**
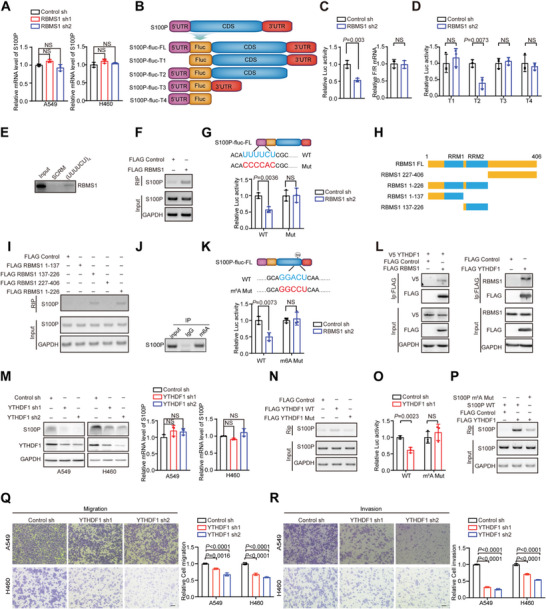
RBMS1 coordinates with YTHDF1 to regulate S100P translation. A) The mRNA level of S100P in A549 and H460 cells with RBMS1 knockdown were examined by RT‐qPCR. *P* values were determined using one‐way ANOVA with Dunnett's multiple comparison test (*n* = 3). B) Schematic of S100P luciferase reporter plasmids: S100P‐fluc‐FL (5′‐UTR, CDS and 3′‐UTR); S100P‐fluc‐T1 (CDS and 3′‐UTR); S100P‐fluc‐T2 (5′‐UTR and CDS); S100P‐fluc‐T3 (5′‐UTR and 3′‐UTR); and S100P‐fluc‐T4 (5′‐UTR). C) H460 cells were cotransfected with S100P‐fluc‐FL luciferase reporter and renilla (loading control), followed by the indicated virus infection of control sh and RBMS1 sh2. Luciferase activity and the mRNA level of S100P‐fluc was examined using RT‐qPCR (*n* = 3). D) H460 cells were cotransfected with S100P‐fluc‐T1, S100P‐fluc‐T2, S100P‐fluc‐T3 or S100P‐fluc‐T4, and renilla (loading control), followed by the indicated virus infection of control sh and RBMS1 sh2. Luciferase activity was measured (*n* = 3). E) Western blot of RBMS1 pulled down with biotin conjugated UUUUCU oligonucleotide or SCRM using cell extracts from HEK293T cells overexpressing RBMS1. F) Binding of S100P 5′‐UTR with RBMS1 was examined by RNA‐IP (RIP) in A549 cells expressing FLAG‐RBMS1. G) Predicted RBMS1‐binding site in 5′‐UTR of *S100P* mRNA in blue and the mutated site in red. H460 cells were cotransfected with S100P‐fluc FL WT or S100P‐fluc FL 5′‐UTR mut, and renilla (loading control), followed by the indicated virus infection of control sh and RBMS1 sh2. Luciferase activity was examined (*n* = 3). H) Schematic of Flag‐RBMS1 full length (1‐406 aa) and truncations (227‐406 aa, 1‐226 aa, 1‐137 aa, 137‐226aa and 137‐406 aa). I) Immunoprecipitation was performed in A549 cells expressing Flag‐RBMS1 full length (1‐406 aa) or truncations (227‐406 aa, 1‐226 aa, 1‐137 aa, 137‐226 aa and 137‐406 aa) and the precipitated were analyzed. J) Anti‐m^6^A IP pulled down S100P mRNA from mRNA of H460 cells using m^6^A antibody with corresponding IgG as controls. RT‐PCR was performed to detect S100P mRNA in elutes. K) Predicted m^6^A site in S100P mRNA by SRAMP program in blue and the mutated site in red. H460 cells were cotransfected with S100P‐fluc‐FL WT or S100P‐fluc‐FL m^6^A mut, and renilla (loading control), followed by the indicated virus infection of control sh and RBMS1 sh2. Luciferase activity was examined (*n* = 3). L) Coimmunoprecipitation was performed in HEK 293T cells expressing FLAG‐RBMS1 or FLAG‐YTHDF1. M) The protein level and mRNA level of S100P were examined in YTHDF1 stable depleted A549 and H460 cells. N) RNA from wild‐type (Flag YTHDF1 WT) and mutant (Flag YTHDF1 Mut) YTHDF1 RIP in A549 cells were measured by RT‐PCR. O) H460 cells were co‐transfected with S100P‐fluc FL WT or S100P‐fluc‐FL m^6^A mut, and renilla (loading control), followed by the indicated virus infection of control sh and R YTHDF1 sh1. Luciferase activity was examined. P) Binding of YTHDF1 with S100P mRNA is m^6^A‐dependent. RIP using YTHDF1 antibody pulled down WT S100P mRNA, but not S100P mRNA with m^6^A site mutated. Q,R) Effect of YTHDF1 knockdown on migration and invasion of A549 and H460 cells evaluated by transwell assays. Scale bars: 100 µm. Data represent mean ± SD, *n* = 3 independent repeats. C,D,G,K,O) *P* values were determined by unpaired Student's *t* test. M,Q,R) *P* values were determined by one‐way ANOVA with Dunnett's multiple comparison test.

To explore how depletion of RBMS1 suppresses the expression of S100P, we first examined the mRNA level of S100P by quantitative real‐time PCR (RT‐qPCR). Interestingly, the mRNA level of S100P was not affected by RBMS1 in both the stably RBMS1‐depleted lung cancer cells and in those with RBMS1 transient knock‐down using siRNA (**Figure** **3**A and Figure S3A,B, Supporting Information), indicating that RBMS1 might regulate the expression of S100P at the post‐RNA level, including translation. We sought to investigate whether RBMS1 influences S100P stability. After treatment with the protein synthesis inhibitor cycloheximide (CHX), RBMS1 depletion had no effect on S100P protein degradation (Figure S3C,D, Supporting Information).

We thus speculated that RBMS1 might regulate the translation of S100P. To test this hypothesis, we performed polysome profiling assay to separate the RNAs into different fractions: non‐translating fraction (<40S), 40S, 60S, 80S monosomes and polysomes from RBMS1 depleted cells and control cells (Figure S3E, Supporting Information). As expected, the S100P mRNA level was significantly decreased in translation‐active polysomes (number of 80S ribosomes ≥ 3) obtained from RBMS1 depleted H460 cells as compared with control H460 cells as judged by RT‐qPCR, while no obvious difference of the GAPDH mRNA level was observed between RBMS1 depleted H460 cells and control H460 cells (Figure S3F, Supporting Information), suggesting that depletion of RBMS1 inhibited S100P translation. To further validate our observation, we conducted luciferase reporter assays using Fluc expression constructs carrying different fragments of S100P: S100P‐fluc‐FL (5′‐UTR, CDS and 3′‐UTR); S100P‐fluc‐T1 (CDS and 3′‐UTR); S100P‐fluc‐T2 (5′‐UTR and CDS); S100P‐fluc‐T3 (5′‐UTR and 3′‐UTR); and S100P‐fluc‐T4 (5′‐UTR) (Figure 3B). Our results demonstrated that knockdown of RBMS1 significantly inhibited the activity of luciferase reporter S100P‐fluc‐FL, but had no significant effect on the mRNA level of S100P‐fluc‐FL (Figure 3C). Moreover, knockdown of RBMS1 only significantly inhibited the activity of luciferase reporter S100P‐fluc‐T2, but not that of S100P‐fluc‐T1, T3 or T4 (Figure 3D and Figure S3G,H, Supporting Information), suggesting that RBMS1 regulates the translation of S100P protein through the 5′‐UTR and CDS region. Further analysis of the S100P sequence revealed an UUUUCU sequence (RBMS1 binding site) in the 5′‐UTR of S100P. A pull‐down assay performed with cell extract efficiently pulled‐down RBMS1 protein with the biotinylated UUUUCU oligonucleotide and not with SCRM oligonucleotide (Figure 3E). RNA‐IP (RIP) assay further confirmed the binding of RBMS1 to the 5′‐UTR of S100P (Figure 3F). Importantly, when we mutated this binding site, S100P‐fluc‐FL luciferase reporter was no longer response to RBMS1 depletion (Figure 3G and Figure S3I, Supporting Information), indicating that RBMS1 recognizes its binding site in the 5′‐UTR of S100P mRNA to stimulate S100P translation.

To further define the region of RBMS1 binding to *S100P* mRNA, we generated four deletion mutants: RBMS1 227‐406 mutant with a deletion of 1‐226 containing RRM1 and RRM2 domains; RBMS1 1‐226 mutant with a C‐terminal deletion; RBMS1 1‐137 mutant containing RRM1 domain; RBMS1 137‐226 mutant only containing RRM2 domain (Figure 3H). By performing an RIP assay, we found that RRM2 domain was responsible for the binding of RBMS1 to S100P mRNA (Figure 3I and Figure S3J, Supporting Information). Meanwhile, we found a potential m^6^A modification site in the CDS near the stop codon of S100P mRNA using SRAMP website (http://www.cuilab.cn/sramp/), which was further verified by MeRIP‐PCR (Figure 3J). When we mutated the m^6^A site, RBMS1 knockdown no longer inhibited the activity of luciferase reporter S100P‐fluc‐FL (Figure 3K and Figure S3K, Supporting Information), indicating that the m^6^A site near the stop codon is also involved in the translation regulation of *S100P* transcripts.

We subsequently investigated how m^6^A modification participated in RBMS1‐regulated translation of *S100P* transcripts. We first used an m^6^A antibody to examine whether RBMS1 affected the m^6^A level in total RNA by dot blot assay and found that RBMS1 did not influence the m^6^A level in total RNA (Figure S4A, Supporting Information). Next, by using a co‐IP assay, we found that RBMS1 interacted with several m^6^A related proteins, including YTHDF1, YTHDF2 and YTHDF3, but not FTO or METTL3 (Figure 3L and Figure S4B, Supporting Information). The expression levels of YTHDF1, YTHDF2 and YTHDF3 were not affected by RBMS1 (Figure S4C, Supporting Information). Importantly, only depletion of YTHDF1, but not YTHDF2 or YTHDF3, decreased the protein level of S100P (Figure 3M and Figure S4D, Supporting Information). However, the mRNA level of S100P was not affected by YTHDF1, as judged by RT‐qPCR in the stably YTHDF1‐depleted H460 and A549 lung cancer cells (Figure 3M, Supporting Information). Similar results were obtained in H460 and A549 cells with YTHDF1 transient depletion using siRNA (Figure S4E,F, Supporting Information). Collectively, our results indicate that YTHDF1 might be involved in RBMS1‐regulated translation of S100P.

We next questioned whether YTHDF1‐regulated S100P expression was dependent on m^6^A modification. Previous study showed that YTHDF1 bound m^6^A sites through its m^6^A‐binding pockets in YTH domain, mutation in K395 and Y397 could abrogate the binding capacity of YTHDF1 with mRNA.^[^ ^27^ ^]^ RNA‐IP assay in HEK293T cells with YTHDF1 overexpression demonstrated that YTHDF1 bound to the CDS of S100P (Figure 3N and Figure S4G, Supporting Information). However, YTHDF1 mutant (YTHDF1‐mut) with K395A and Y397A mutations significantly decreased the binding capacity to S100P (Figure 3N and Figure S4G, Supporting Information). Additionally, we constructed a mutant reporter of S100P‐fluc‐FL with m^6^A site mutation by replacing the adenosine bases in m^6^A sites with cytosine (S100P‐fluc‐FL m^6^A mut). The luciferase activity was significantly reduced in cells transfected with wild‐type S100P‐fluc‐FL, but not in cells transfected with S100P‐fluc‐FL m^6^A mut, upon YTHDF1 knockdown as compared to control cells (Figure 3O and Figure S4H, Supporting Information). The RNA‐IP assay revealed that YTHDF1 could bind to wild‐type S100P‐fluc‐FL, but its binding to S100P‐fluc‐FL m^6^A mut was significantly decreased (Figure 3P and Figure S4I, Supporting Information), further supporting that YTHDF1‐regulated S100P translation was dependent on its m^6^A recognition function. We next explored the role of YTHDF1 in lung cancer metastasis. Transwell migration and invasion assays showed that YTHDF1 knockdown significantly decreased the migration and invasion ability of A549 and H460 cells compared with control cells (Figure 3Q,R). Taken together, our results suggest that RBMS1 coordinates with YTHDF1 to regulate S100P translation through S100P 5′‐UTR and m^6^A modification.

### 2.5 RBMS1 Interacts with YTHDF1 to Bridge the 5′‐UTR and CDS of S100P to Promote Its Translation

**Figure 4:**
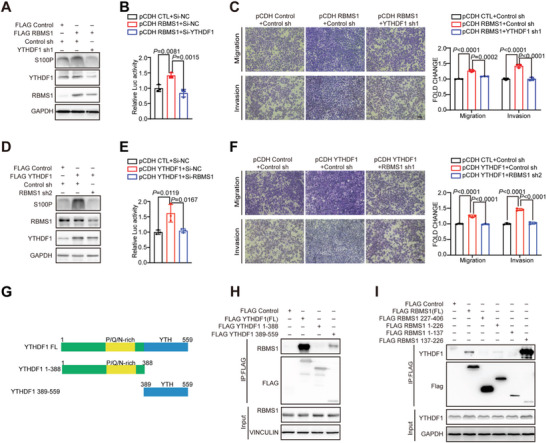
RBMS1 interacts with YTHDF1 to bridge the 5′‐UTR and CDS of S100P to promote its translation. A) The protein level of S100P, RBMS1, and YTHDF1 were examined in A549 cells expressing RBMS1 with or without YTHDF1 depletion. B) The luciferase activity of S100P‐fluc‐FL was examined in H460 cells expressing RBMS1 with or without YTHDF1 depletion. *P* values were determined by one‐way ANOVA with Tukey's multiple comparison test (*n* = 3). C) Effect of YTHDF1 knockdown on migration and invasion of A549 overexpressing RBMS1 cells evaluated by transwell assays. Scale bars: 100 µm. *P* values were determined by one‐way ANOVA with Tukey's multiple comparison test (*n* = 3). D) The level of S100P, RBMS1 and YTHDF1 were examined in A549 cells expressing YTHDF1, with or without RBMS1 depletion. E) The luciferase activity of S100P‐fluc‐FL was examined in H460 cells expressing YTHDF1 with or without RBMS1 depletion. *P* values were determined by one‐way ANOVA with Tukey's multiple comparison test (*n* = 3). F) Effect of RBMS1 knockdown on migration and invasion of A549 expressing YTHDF1 cells evaluated by transwell assays. Scale bars: 100 µm. *P* values were determined by one‐way ANOVA with Tukey's multiple comparison test (*n* = 3). G) Schematic of Flag‐YTHDF1 full length (1‐559 aa) and truncations (1‐388 aa and 388‐559). H) Immunoprecipitation was performed in HEK 293T cells expressing Flag‐YTHDF1 full length (1‐559 aa) or truncations (1‐388 aa and 388‐559 aa) and the precipitated were analyzed. I) The deletion‐mapping assay showed that RRM2 domain (137‐226 aa) of RBMS1 bound to YTHDF1.

Based on the above results, we hypothesized that YTHDF1 may serve as a functional coregulator with RBMS1 to mediate the translation of S100P mRNA. To investigate whether YTHDF1 is required for RBMS1‐regulated translation of S100P, we co‐transfected cells with RBMS1 together with either an empty vector or YTHDF1 shRNA and then measured the protein level of S100P using a western blot assay. We found that overexpression of RBMS1 significantly increased the protein level of S100P, however RBMS1‐induced upregulation of S100P protein level was abolished by YTHDF1 depletion (**Figure** **4**A). Similar results were also obtained in the S100P‐fluc‐FL reporter assay (Figure 4B). Further transwell assays showed that depletion of YTHDF1 partially reversed the RBMS1‐induced elevated cell migration and invasion in A549 cells (Figure 4C). Meanwhile, overexpression of YTHDF1 significantly increased the protein level of S100P, however YTHDF1‐induced upregulation of S100P protein level was abolished by RBMS1 depletion (Figure 4D). Similar results were validated using S100P‐fluc‐FL reporter (Figure 4E). Consistent with this, overexpression of YTHDF1 promoted cell migration and invasion, whereas YTHDF1 induced cell migration and invasion was suppressed by RBMS1 knockdown (Figure 4F).

We further generated various truncated vectors of both YTHDF1 and RBMS1 to investigate their interacting regions, and found that the binding between RBMS1 and YTHDF1 is dependent on the RRM2 domain in RBMS1 and the YTH domain in YTHDF1 (Figure 4G–I).

### 2.6 RBMS1 Is Positively Correlated to the Level of S100P in Clinical Samples

**Figure 5:**
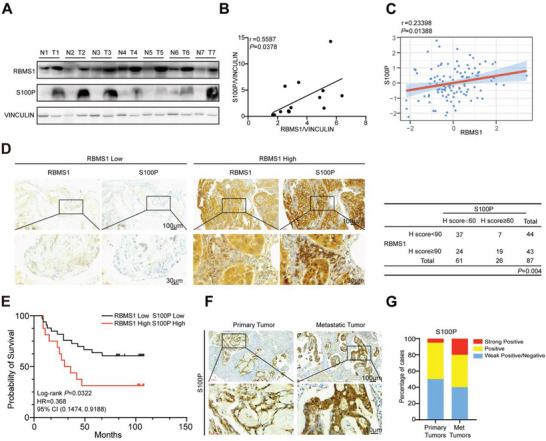
RBMS1 positively correlates with S100P in clinical samples. A) RBMS1 and S100P levels of 7 paired lung cancer patient tumors (T) and adjacent tissues (N) were analyzed. B) Correlation of RBMS1 with S100P levels was analyzed. C) Correlation of RBMS1 and S100P levels was analyzed using the data of human LUAD samples and normal tissues in the CPTAC data portal. D) Representative images of RBMS1 and S100P immunohistochemical staining in lung tumor specimens (*n* = 90). Scale bars: 100 µm (top) and 30 µm (bottom). The correlation between RBMS1 and S100P expression was calculated by Pearson's chi‐square test. E) Kaplan‐Meier curve showing overall survival of NSCLC patients with high RBMS1 expression and high S100P expression or low RBMS1 expression and low S100P expression (*P* values were determined by log‐rank test). F) Representative images from immunohistochemical staining of S100P in metastatic lymph node (*n* = 30) and matched primary lung adenocarcinoma tissues (*n* = 30). Scale bars: 100 µm (top) and 30 µm (bottom). G) The quantification of S100P protein level in metastatic lymph node and a primary lung adenocarcinoma tissue. The S100P levels were classified into 3 grades (weak positive/negative, positive, strong positive) based on quantification of immunohistochemical staining and plotted.

To investigate the clinical relevance of RBMS1 and S100P in lung cancer, we examined the protein levels of RBMS1 and S100P in fresh‐frozen tumor and paired adjacent normal tissues from 7 patients with NSCLC. Tumor samples with high expression of RBMS1 exhibited increased S100P levels and the level of S100P was positively correlated to that of RBMS1 in clinical samples (*R* = 0.5587, *P* < 0.0378; **Figure** **5**A,B). Consistently, we also analyzed the data of human LUAD samples and normal tissues in the CPTAC data portal, and further Spearman's rank correlation analysis proved a significant and positive correlation between RBMS1 and S100P (Figure 5C). Similar results were obtained by immunohistochemistry analysis using human lung tissue microarray containing 90 tumor tissue and adjacent normal tissue samples (Figure 5D). Most importantly, lung cancer patients with high expression of RBMS1 and S100P had a worse prognosis than those with low expression of RBMS1 and S100P (Figure 5E). We further determined S100P expression using a tissue microarray consisting of 30 primary lung cancer tissues and matched metastatic tissues. The IHC results showed that the expression level of S100P was higher in most metastatic tumors than that in the matched primary tumors (Figure 5F,G). Collectively, our clinical data reveal that RBMS1 is positively correlated with S100P in lung cancer patient samples.

### 2.7 Nortriptyline Hydrochloride (NTP), a Small‐Molecule Inhibitor of RBMS1, Attenuates Tumor Metastasis by Inhibiting S100P Expression

**Figure 6:**
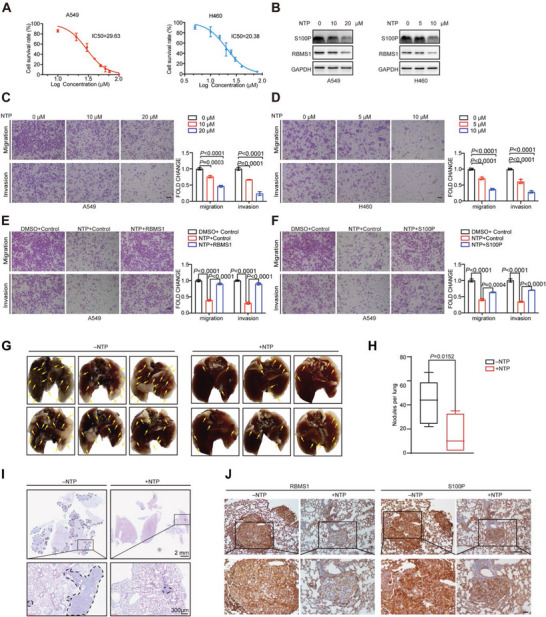
RBMS1 small‐molecule inhibitor Nortriptyline hydrochloride (NTP) attenuates tumor metastasis by inhibiting S100P expression. A) IC50 of NTP in A549 and H460 cells were measured by CCK8 assay. B) S100P and RBMS1 protein level in A549 and H460 cells treated with gradient concentration of NTP were examined. C) The migration and invasion of A549 cells treated with gradient concentration of 0 × 10^−6^, 10 × 10^−6^, 20 × 10^−6^ m NTP was measured by transwell assay. *P* values were determined using one‐way ANOVA with Dunnett's multiple comparison test (*n* = 3). Scale bars: 100 µm. D) The migration and invasion of H460 cells treated with gradient concentration of 0 × 10^−6^, 5 × 10^−6^, 10 × 10^−6^ m NTP was measured by transwell assay. *P* values were determined using one‐way ANOVA with Dunnett's multiple comparison test (*n* = 3). Scale bars: 100 µm. E) Effect of RBMS1 overexpression on migration and invasion of A549 cells treated with 20 × 10^−6^ m NTP was detected by transwell assays. *P* values were determined by one‐way ANOVA with Tukey's multiple comparison test (*n* = 3). Scale bars: 100 µm. F) Effect of S100P overexpression on migration and invasion of A549 cells treated with 20 × 10^−6^ m NTP was detected by transwell assays. *P* values were determined by one‐way ANOVA with Tukey's multiple comparison test (*n* = 3). Scale bars: 100 µm. G) Effect of NTP on tumor metastasis potential in A549 cells was examined by tail vein injection metastasis model. H) The number of lung metastatic nodules in all five lung lobes from each mouses was counted and statistically analyzed. (*n* = 5 for each group). *P* values were determined by unpaired Student's *t* test. I) The pulmonary metastases in the mouse model were histologically analyzed by H&E staining. Scar bars, 4 mm (left). Representative lung metastatic nodules are shown. Scar bars, 300 µm (right). J) Lungs was removed from mice treated with or without NTP and subjected to immunohistochemical staining with anti‐RBMS1 and anti‐S100P antibody. Scale bars: 100 µm.

Our finding that RBMS1 is upregulated in metastatic lung cancer and depletion of RBMS1 suppresses tumor metastasis identifies RBMS1 as an attractive anti‐metastatic target. We applied a previously identified RBMS1 inhibitor, NTP,^[^ ^15^ ^]^ to treat A549 and H460 cells. We performed CCK8 assay to determine the IC50 of NTP in A549 and H460 cells. The IC50 values of A549 and H460 cells were 29.63 × 10^−6^ and 20.38 × 10^−6^ m respectively (**Figure** **6**A). Intriguingly, we found that NTP treatment reduced the levels of RBMS1 and S100P in both A549 and H460 lung cancer cells in a dose‐dependent manner (Figure 6B). Moreover, to determine the effect of NTP on NSCLC migration and invasion, we treated A549 cells with NTP at 0 × 10^−6^, 10 × 10^−6^, 20 × 10^−6^ m, and H460 cells with NTP at 0 × 10^−6^, 5 × 10^−6^, 10 × 10^−6^ m. The cell migration and invasion were inhibited by NTP in a dose‐dependent manner, as judged by transwell migration and invasion assays (Figure 6C,D). To further verify that NTP therapy for lung cancer is via the RBMS1/YTHDF1/S100P signaling axis, we re‐expressed RBMS1 in A549 cells with NTP treatment and found that restoration of RBMS1 almost fully reversed the NTP‐induced inhibition of cell migration and invasion, as judged by transwell migration and invasion assays (Figure 6E and Figure S5A, Supporting Information). Similarly, S100P re‐expression also reversed the NTP‐induced inhibition of cell migration and invasion (Figure 6F and Figure S5B, Supporting Information). Therefore, these data further verified that NTP therapy for lung cancer is via the RBMS1/YTHDF1/S100P signaling axis.

Importantly, treatment with the dose of 20 mg kg^−1^ NTP every 2 d significantly attenuated metastatic potential in a xenograft mouse model inoculated with A549 cells (Figure 6G,H). H&E staining of excised lung sections confirmed that NTP group had smaller and fewer lung metastatic foci than those in the control group (Figure 6I). IHC assay further verified that levels of RBMS1 and S100P were decreased upon NTP treatment (Figure 6J). Altogether, our data indicate that RBMS1 is a promising anti‐metastasis target, and the RBMS1 inhibitor NTP is a potent therapeutic agent for anti‐metastasis therapy in human lung cancer.

## 3 Discussion

**Figure 7:**
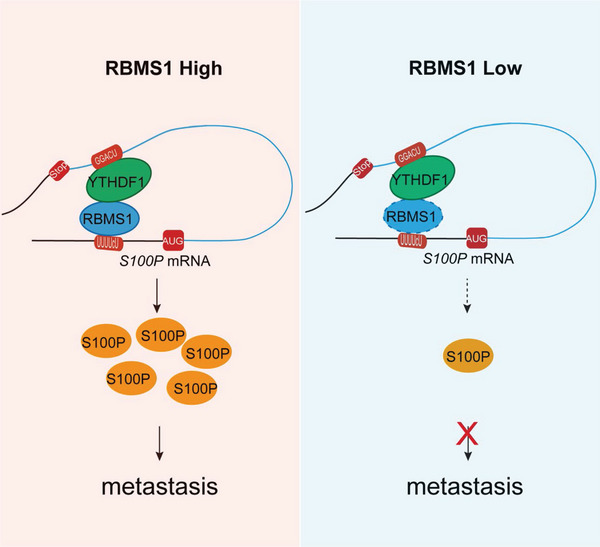
Schematic of how RBMS1 coordinates with YTHDF1 to regulate S100P translation and metastasis in lung cancer.

Metastasis is responsible for the main cause of lung cancer‐related death. Metastasis is a complex process that requires the regulation of multiple cellular signaling pathways and functions. Nevertheless, the underlying molecular mechanisms of lung cancer remain largely unknown. In the present study, we found that RBMS1 is highly expressed in metastatic NSCLC tissues and significantly associated with shorter survival time. Moreover, modulation of RBMS1 expression revealed its oncogenic activity through promoting cell migration and invasion. Consistently, in vivo studies using a mouse model revealed that RBMS1 plays a critical role in tumor metastasis. Most importantly, we found a novel molecular mechanism by which RBMS1 interacts with YTHDF1 to promote S100P translation, thereby stimulating NSCLC metastasis (**Figure** **7**). Therefore, RBMS1 may be a potential therapeutic target for strategies designed to inhibit NSCLC metastasis. We then applied NTP, an RBMS1 inhibitor, to target RBMS1, and found that NTP attenuated tumor metastasis both in vitro and in vivo studies using a mouse lung metastasis model. Taken together, our study revealed that RBMS1 is a promising anti‐metastasis target and the RBMS1 inhibitor NTP is a potent therapeutic agent for anti‐metastasis therapy in human lung cancer.

Previously, RBMS1 was reported as a suppressor of metastasis through targeted stabilization of its mRNA regulon in colon cancer.^[^ ^18^ ^]^ However, the role of RBMS1 in NSCLC metastasis has not been characterized yet. Surprisingly, we revealed that RBMS1 was upregulated in metastatic NSCLC and high RBMS1 expression had a significantly shorter survival time and poor prognosis. A series of functional experiments in vitro and in vivo confirmed that knockdown of RBMS1 inhibited cell metastasis in NSCLC, indicating that RBMS1 is involved in NSCLC metastasis as a tumor promoter. Our results reveled that the role of RBMS1 in NSCLS metastasis is different from that in colon cancer. We proposed that these findings may be due to tissue specificity and tumor genotype, which is similar to the distinct roles of PGC1α in different cancers,^[^ ^28^, ^29^ ^]^ but the underlying specific molecular mechanisms remain to be further explored.

RBMS1 has been reported to regulate DNA replication, DNA transcription, and RNA stability.^[^ ^16^, ^17^, ^18^ ^]^ Recently, we revealed that RBMS1 could modulate the translation of SLC7A11 by binding to its 3′‐UTR.^[^ ^15^ ^]^ When RBMS1 is depleted, the translation of SLC7A11 was inhibited, and the reduced SLC7A11 promoted ferroptosis, thereby inhibiting lung cancer proliferation in cultured cancer cells, xenograft mice, and genetically conditional knockout mice.^[^ ^15^ ^]^ However, the function of RBMS1 in NSCLC metastasis is less understood. We observed that RBMS1 is highly expressed in metastatic NSCLC tissues. Functional study of RBMS1 revealed that RBMS1 promotes cell migration and invasion. Further mechanistic study revealed RBMS1 could recognize its binding site in the 5′‐UTR of S100P mRNA to stimulate S100P translation, thereby stimulating NSCLC metastasis.

Importantly, we identified YTHDF1 as a coregulator of RBMS1 in mediating the translation of S100P transcripts. Methylation of adenosine nucleotides at the N6 position (m^6^A) is the most abundant posttranscription modification in mammalian mRNA, which is reversible through a set of enzymatic reactions.^[^ ^30^ ^]^ YTHDF1 is one of three major m^6^A “reader” proteins that have been shown to recognize m^6^A nucleotides via their YTH (YT521‐B homology) domain.^[^ ^31^, ^32^ ^]^ YTHDF1 has been reported to promote translation by interacting with translation machinery.^[^ ^33^ ^]^ We confirmed the putative interaction between RBMS1, YTHDF1 and the S100P transcript using a series of rescue experiments in A549 cells. Importantly, S100P m^6^A site mutation by replacing the adenosine bases in m^6^A sites with cytosine abolished the translational regulation by RBMS1 or YTHDF1. Mechanistically, our data suggest that RBMS1 binds to the 5′‐UTR of S100P transcript via its RRM2 domain and YTHDF1 binds to the m^6^A site of S100P transcript via its YTH domain. In addition, RRM2 domain of RBMS1 and the YTH domain of YTHDF1 are required for the binding of RBMS1 and YTHDF1.

In conclusion, RBMS1 coordinates with YTHDF1 to regulate S100P translation in an m^6^A dependent manner. Moreover, our findings that RBMS1 expression positively correlates with lung cancer metastatic progression and RBMS1 knockdown attenuates tumor metastasis, suggest that RBMS1 may represent an attractive anti‐metastatic target for lung cancer treatment. Compound NTP, as RBMS1 inhibitor, has shown promising efficacy in the treatment of lung cancer cells and in mouse models of tumor metastasis. Targeting RBMS1 has the potential to be an effective treatment strategy for lung cancer patients.

## 4 Experimental Section

### Cell Culture

All cell lines used in this study were purchased from the American Type Culture Collection (ATCC). HEK293T cells were cultured in DMEM medium supplemented with 10% FBS. A549 cells were cultured in F12K medium supplemented with 10% FBS. NCI‐H460 and H2170 cells were cultured in RPM‐1640 medium supplemented with 10% FBS. All cell lines were cultured in a humid environment of 37 °C with 5% CO_2_.

### Plasmid Constructions

For shRNA plasmids used in lentivirus‐mediated interference, complementary sense and antisense oligonucleotides encoding shRNAs targeting RBMS1 or YTHDF1 were synthesized, annealed and cloned into pLKO.1 vector or pLKO‐Tet‐On inducible vector. The full length of human RBMS1 and S100P cDNA were cloned into the lentivirus vector pCDH‐CMV‐MCS‐EF1‐Puro with N‐terminal Flag tag with restriction enzymes Nhe I and Not I. Flag‐YTHDF1 and mutation plasmids Flag‐YTHDF1‐K395A/Y397A was constructed via cloning YTHDF1 with Flag tag into pCDH‐CMV‐MCS‐EF1‐Puro vector. The truncated plasmid was generated based on the full‐length cDNA of RBMS1 and YTHDF1. To generate S100P luciferase reporter, the DNA fragment of human *S100P* gene 5′‐UTR was amplified by PCR and cloned into Hind III site of the PGl3‐control‐vector (S100P‐fluc‐5′‐UTR). Then the DNA fragment of human *S100P* gene CDS and 3′‐UTR was amplified by PCR and cloned into Xba I site of the PGl3‐control‐vector. All construction were confirmed by DNA sequencing. Primers for PCR amplification, shRNAs are listed in Table S2 (Supporting Information).

### Cell Line Generation

To generate RBMS1 stable‐knockdown cell lines, the pLKO.1 RBMS1 constructs or pLKO.1‐Tet‐on‐RBMS1‐shRNA was transfected into HEK293T cells together with pPAX2 and pMD2 lentiviral packaging systems using LipoPlus reagent. After 72 h, the viruses were collected and then used to infect A549, NCI‐H460 and H2170 cells with Polybrene (8 µg mL^−1^) for 24 h. Then, stably integrated cells were selected with puromycin (5 µg mL^−1^) for 5 d. After that, stable cell lines were maintained in medium containing 2 µg mL^−1^ puromycin. A549 and H460 were infected by lentiviral pCDH‐Flag RBMS1 to generate stable lines with RBMS1 overexpression. RBMS1 expression was determined by western blot analysis. Cells transfected with lentivirus with empty vectors were used as controls.

To generate GFP labeled RBMS1 knockdown A549 cells and mCherry labeled control A549 cells, mCherry or GFP stable‐expressed A549 cell lines were first generated. Briefly, the pCDH‐MCS‐EF1‐Hygro‐mCherry/GFP constructs were transfected into HEK 293T cells together with pPAX2 and pMD2 lentiviral packaging systems using LipoPlus reagent. After 72 h, the viruses were collected and then used to infect A549, with Polybrene (8 µg mL^−1^) for 24 h. Subsequently, stably integrated cells were selected with hygromycin (500 µg mL^−1^) for 5 d. Then the transfected pool was diluted for single clone selection. The clone expressing mCherry or GFP fluorescence was selected, and the cell line was established from one single clone. Then, lentivirus with pLKO.1 RBMS1 was used to infect GFP stable‐expressed A549 cell lines to generate GFP labeled RBMS1 knockdown A549 cells, and lentivirus with pLKO.1 empty vectors was used to infect mCherry stable‐expressed A549 cell lines to generate mCherry labeled control A549 cells.

### Cell Migration and Invasion Assays

Cell migration and invasion were evaluated by using the transwell assay. Chambers were present in a 24‐well culture table with 600 µL complete medium containing 20% FBS prepared at the bottom. Then, 5 × 10^4^ A549 or 1 × 10^5^ H460 cells were suspended in 100 µL of serum‐free medium and loaded into 8.0 µm pore size polycarbonate membrane chamber coated with (invasion assay) or without (migration assay) Matrigel (BD Biosciences), in triplicates. Cells were further incubated at 37 °C with 5% CO_2_. Then the chamber was fixed with 4% paraformaldehyde and stained with 0.5% crystal violet. Non‐migrating cells in the inner layer were removed with cotton swab. Cells on the bottom surface were imaged under a standard bright field microscope (10× objective) equipped with a digital camera.

### Gelatin Degradation Assay

To measure the ability of cells to form invadopodia and degrade matrix, QCM Gelatin Invadopodia Assay (ECM670, Merck Millipore) was performed according to the manufacturers’ protocol. Briefly, the 96‐well plates were pretreated with 0.2% w/v poly‐L‐lysine for 20 min at room temperature and then fixed with 0.5% glutaraldehyde at room temperature for 15 min. FITC‐gelatin and unlabeled gelatin were mixed at 1:5 and added to 96‐well plates. After incubating at room temperature for 10 min, the 96‐well plates were disinfected with 70% ethanol. The 96‐well plates were washed three times with PBS. Then, 2 × 10^3^ A549 cells were suspended in medium with 10% FBS and seeded into the 96‐well plate. After 36 h, remove the medium, the cells were washed twice with PBS. After 30 min fixation with 3.7% formaldehyde, staining was respectively performed with TRITC‐phalloidin and DAPI for 1 h. The ability of cells to degrade the matrix was imaged using immunofluorescence microscopy (LEICA DMi8) and the area of invadopodia was measured using the Image J software.

### 3D Matrix Multicellular Spheroids Model

3D matrix multicellular spheroids model previously reported^[^ ^26^ ^]^ was used to measure the cell invasion ability. GFP labeled RBMS1 knockdown A549 cells and mCherry labeled control A549 cells were constructed. Before cell seeding in the poly‐dimethylsiloxane (PDMS, Sylgard 184, Dow Corning, USA) hemispheric microwell, the surface of the device was immersed in 1% PluronicF‐127 (Sigma‐Aldrich) solution for 4 h and then washed twice with PBS. Equal amount of A549 control sh and A549 RBMS1 sh1 cells were mixed, centrifuged, and resuspended at a density of 2.5 × 10^7^. 200 µL cell suspension were seeded in the microwell and the microchip was placed in humidified air with 5% CO_2_ at 37 °C for 48 h to allow for multicellular spheroid formation. Spheroids were collected and resuspend with 450 µL collagen I (BD Biosciences, Bedford, MA, USA) solution at a diluted working concentration 4 mg mL^−1^. Then, 150 µL collagen solution containing spheroids were added into the 48‐well plate. After crosslinking and solidifying the collagen mixture at 37 °C for 30 min, 300 µL medium was added into each well. Cultures were maintained by replacing the medium every day. After 96 h, the tumor spheroids in the collagen matrix were fixed with 4% paraformaldehyde. Fluorescent photographs were taken using a confocal laser scanning biological microscope (Olympus FV1000, Japan).

### Tail Vein Injection Metastasis Model

The experimental protocol on animals was approved by the Institutional Animal Care and Use Committee of Dalian Medical University. To identify the RBMS1 function on lung cancer metastasis, a tail vein injection experiment in BALB/c Nude mice was performed. Briefly, nude mice with 8 weeks old were randomly divided into three groups and each mouse was injected through tail vein with 2 × 10^6^ A549 cells with Control sh, RBMS1 sh1or RBMS1 sh2. The mice were weighed every 2 d to detect their health status. After 13 weeks, the mice were euthanized, and the lung tissues were fixed with 4% formaldehyde after the lungs were removed. The lung tissues were embedded in paraffin for further analysis.

To determine the role of NTP in vivo, 3.5 ×10^6^ A549 WT cells were injected into nude mice through tail vein. After 1 week, the mice were assigned randomly into two treatment groups. NTP was dissolved in dimethyl sulfoxide (DMSO) and diluted in PBS. NTP was intraperitoneally injected into mice at a dose of 20 mg kg^−1^ every 2 d. The other group mice were injected equivalent PBS. After 8 weeks, the mice were euthanized, and lung tissues were removed and fixed with 4% formaldehyde. The lung tissues were dehydrated, waxed immersion and embedded. And Paraffin‐embedded lung tissue specimens were sectioned for the H&E staining.

### Immunohistochemical Staining

The human lung cancer tissue microarrays were purchased from Shanghai Outdo Biotech, China. The tissue sections were deparaffinized and rehydrated. The sections were microwave heated in 0.01 m sodium citrate (pH 6.0) for antigenic repair. A drop of 3% H_2_O_2_ was added to the tissue to suppress endogenous peroxidase activity. The sections were covered with 3% goat serum for blocking. Then the sections were incubated with primary antibody at dilution of 1:100 (Anti‐RBMS1 antibody) and 1:2000 (Anti‐S100P antibody) at 4 °C overnight. The sections were incubated with biotin‐labeled goat anti‐mouse IgG. Next, the sections were incubated with Horseradish peroxidase labeled Streptomycin. Slides were stained with 3,3′‐diaminobenzidine. Finally, the sections were washed, counterstained with hematoxylin, dehydrated, treated with xylene, and sealed with neutral resin.

### Immunoprecipitation

HEK293T cells were transfected with pCDH‐Flag‐RBMS1 (pCDH‐Flag‐YTHDF1) or empty vector for 24 h using Sage Lipoplus Reagent according to the certificate of manufacturer. Cells were lysed with IP lysis buffer containing 50 × 10^−3^ m Tris‐HCl (7.4), 150 × 10^−3^ m NaCl, 1  × 10^−3^ m EDTA, 1  × 10^−3^ m PMSF, 1×Cocktail, 1% TritonX‐100 for 30 min. The cell lysate was centrifuged at 12 000 rpm for 15 min. The supernatant was subjected to immunoprecipitation using anti‐Flag M2 Affinity Gel (Sigma Aldrich) at 4 ˚C overnight. Next day, the samples were washed five times with 1 mL washing buffer (50 × 10^−3^ m Tris‐HCl pH 7.5, 150 × 10^−3^ m NaCl, 0.5% TritonX‐100, 10% Glycerol). The samples were eluted with 100 µL Flag peptide (100 µg mL^−1^) at 4 ˚C for half an hour. The eluted protein samples were subjected to western blotting.

### Western Blot

Cells were washed twice with cold PBS. Then the cells were lysed with RIPA lysis buffer (50 × 10^−3^ m Tris‐HCl (pH 7.4), 150 × 10^−3^ m NaCl, 1% Triton X‐100, 1% sodium deoxycholate, 0.1% SDS, sodium orthovanadate, sodium fluoride, EDTA, leupeptin) for 30 min. The cell lysate was centrifuged at 12 000 rpm for 15 min. The protein concentration was measured using BCA protein assay kit. Equal amounts of total protein were separated on 10% or 15% SDS‐PAGE and transferred onto NC membranes, blocked with 5% nonfat milk at room temperature for 1 h, and incubated with primary antibodies at 4 ˚C overnight. After washed three times, the membranes were incubated with secondary Ig conjugated HRP for 1 h at room temperature. Protein bands were visualized with the ECL enhanced chemiluminescence regent Kit (NCM Biotech). The following antibodies were used: RBMS1 (Abcam ab150353), S100P (Proteintech 11803‐1‐AP), YTHDF1 (Proteintech 17479‐1‐AP), YTHDF2 (Proteintech 24744‐1‐AP), YTHDF3 (Proteintech 25537‐1‐AP), METTL3 (Proteintech 15073‐1‐AP), METTL14 (Proteintech 26158‐1‐AP), FTO (Proteintech 27226‐1‐AP), m6A (Proteintech 68055‐1‐lg), FLAG (Sigma 1804), V5 (Proteintech 14440‐1‐AP), VINCULIN (Proteintech 66305‐1‐Ig), GAPDH (Proteintech 60004‐1‐Ig).

### Me‐RIP

Total RNA from cultured H460 cells was isolated using Trizol (Invitrogen) according to the manufacturers’ instructions. The extracted total RNA was treated with DNase I (QIAGEN RNeasy Mini Kit) to remove gDNA. Immunoprecipitation of m^6^A‐modified RNAs was performed as previously described.^[^ ^34^ ^]^ Briefly, the total RNA was fragmented into small fragments using RNA Fragmentation Reagents (AM8740, ThermoFisher, USA). The fragmented RNA was incubated with m^6^A‐specific antibody (Cat No. 68055‐1‐Ig, Proteintech) in IP buffer (10 × 10^−3^ m Tris‐HCl pH7.4, 150 × 10^−3^ m NaCl, 0.1% NP40, 1U mL^−1^ RNase inhibitor) for 2 h at 4 °C. The mixture was then incubated with protein A beads (Thermo Fisher Scientific) that had been pre‐blocked with BSA for 2 h on a rotating wheel at 4 °C. The beads were washed three times with IP buffer. The bound RNA was eluted from the beads with 100 µL Elution buffer (10 × 10^−3^ m Tris‐HCl pH 7.4, 150 × 10^−3^ m NaCl, 0.1% NP40, 1U mL^−1^ RNase inhibitor and 6.7 × 10^−3^ m m^6^A), followed by ethanol precipitation. Purified RNA was reverse transcribed and tested by PCR.

### m^6^A Dot Blot

Total RNA was isolated using Trizol (Invitrogen) according to the manufacturers’ instructions. The equal amounts RNA was denatured in 10 µL RNA incubation buffer (50% formamide, 2.5% formaldehyde and 0.5× MOPS) at 55 °C for 15 min, followed by chilling on ice. The denatured RNA was plated on Amersham Hybond‐N+ membrane. The membranes were crosslinked using 70 000 µj cm^−2^ UV. After crosslinking, the membranes were stained by 0.02% MB (methylene blue). Then the membranes were washed with PBST, blocked 5% milk for 1 h and incubated with m^6^A antibody (Proteintech 68055‐1‐Ig) at 4 ˚C overnight. After washed three times, the membranes were incubated with secondary Ig conjugated HRP for 1 h at room temperature. The membranes were visualized with the ECL enhanced chemiluminescence regent Kit (NCM Biotech).

### RNA Pulldown

Streptavidin beads (ThermoFisher Scientific Cat:11205D) were washed with 1 mL lysis buffer (10 × 10^−3^ m HEPES pH7.0, 200 × 10^−3^ m NaCl,10 × 10^−3^ m MgCl_2_, 1 × 10^−3^ m DTT, 1% Triton X‐100) for twice. The beads were blocked with 1 mL lysis buffer containing 5% BSA and 100 µg tRNA at 4 °C for 1 h. HEK293T cells with stable expressing Flag‐RBMS1 were lysed in 1 mL lysis buffer (containing 1 × 10^−3^ m PMSF and 1 × Cocktail) and subjected to three rounds of sonication. The cell lysate was precleared by mixing with washed streptavidin beads along with nonspecific tRNA to get rid of nonspecific binding at 4 °C for 1 h. 800 pmol of synthetic Biotin‐RNAs were denatured at 65 °C for 5 min then cooled to room temperature in the presence of 5× RNA structure buffer (50 × 10^−3^ m HEPES pH7.0, 50 × 10^−3^ m MgCl_2_). Pre‐cleared cell lysate was incubated with 5 µL RNasin (40 U µL^−1^) and denatured RNAs on a rotating shaker at 4 °C for 1 h. The RNA tethered cell lysate was added into the blocked Streptavidin beads and incubated at 4 °C for 3 h. After incubation, beads were washed with 1 mL washing buffer (10 × 10^−3^ m HEPES pH7.0, 0.4 m NaCl,10 × 10^−3^ m MgCl_2_, 1 × 10^−3^ m DTT, 1% Triton X‐100, 4 U mL^−1^ RNasin, 1 × 10^−3^ m PMSF and 1 × Cocktail) for six times. The bound proteins were subjected to western blotting.

### RNA Immunoprecipitation

RNA immunoprecipitation was carried out as previously described. Briefly, Flag‐RBMS1 (Falg‐YTHDF1) or control A549 cells (HEK 293T) were collected and cross‐linked with 1% formaldehyde for 10 min. Then the samples were blocked by glycine solution (the final concentration is 0.25 m) for 5 min and washed twice with cold PBS. The cells were collected and lysed by 1 mL IP lysis buffer (50 × 10^−3^ m Tris‐HCl pH 7.5, 0.4 m NaCl, 1 × 10^−3^ m EDTA, 1 × 10^−3^ m DTT, 0.5% TritonX‐100, 10% Glycerol containing protease inhibitors and RNase inhibitor) and subjected to three rounds of sonication. The cell lysate was precleared by mixing with Protein A/G agarose beads along with nonspecific tRNA to get rid of nonspecific binding and collected for immunoprecipitation with Anti‐Flag M2 Affinity beads. After six times washes with 1 mL of IP lysis buffer (50 × 10^−3^ m Tris‐HCl pH 7.5, 0.4 m NaCl, 1 × 10^−3^ m EDTA, 1 × 10^−3^ m DTT, 0.5% TritonX‐100, 10% Glycerol containing protease inhibitors and RNase inhibitor), the beads were resuspended in 100 µL RIP buffer (50 × 10^−3^ m Tris‐HCl pH 7.5, 0.1 m NaCl, 5 × 10^−3^ m EDTA, 10 × 10^−3^ m DTT, 0.5% TritonX‐100, 10% Glycerol, 1% SDS) and incubated at 70 °C for 45 min to reverse the crosslinks. The RNA was extracted using Trizol and reverse transcribed into cDNA for PCR detection.

### RNA Isolation and RT‐qPCR

Total RNA was extracted from target cells or RNA‐IP samples using Trizol reagent (Invitrogen) according to the manufacturer's instructions. 1 µg of mRNA was used to synthesize cDNAs by using Hifair II 1st Strand cDNA Synthesis SuperMix (Yeasen, China) with random primer and qPCR reactions were carried out using the Maxima SYBR Green qPCR Master Mix (Thermo Scientific). Transcripts of the housekeeping gene GAPDH in the same incubations were used for normalization. The Gene relative expression levels were calculated using the 2^−ΔΔCT^ method.

### Polysome Profiling

The H460 cells with or without RBMS1 knockdown were treated with 100 µg mL^−1^ cycloheximide (CHX) at 37 °C for 5 min. The cells were washed with ice‐cold PBS solution containing 100 µg mL^−1^ cycloheximide (CHX) and lysed in polysome lysis buffer (5 × 10^−3^ m Tris‐HCl pH 7.5, 2.5 × 10^−3^ m MgCl_2_, 1.5 × 10^−3^ m KCl,100 µg mL^−1^ CHX, 1× protease inhibitor cocktail, 100 U mL^−1^ RNase inhibitor, 25 U mL^−1^ DNase I, 2 × 10^−3^ m DTT, 0.5% TritonX‐100 and 0.5% sodium deoxycholate), followed by centrifugation at 12 000 *g* for 10 min at 4 °C. The lysate was applied to sucrose gradients buffer of 10%−50% and centrifuged at 35 000 rpm for 2 h at 4 °C. Fractions were collected using a density gradient fractionation system (Brandel). Total RNA from each fraction was isolated using Trizol (Invitrogen) according to the manufacturers’ instructions. The mRNA level of S100P and GAPDH in each fraction were examined by RT‐qPCR.

### Luciferase Assay and Translation Efficiency

The NCI‐H460 cells were cotransfected with S100P luciferase reporters with renilla control plasmid for 24 h. Then the cells were plated in 24‐well plates and the following day added the viruses of RBMS1 sh (YTHDF1 sh) or Control sh. After 48 h, the luciferase activities were measured following dual luciferase reporter assay detection kit (Promega Corporation, USA). The parallel group cells were collected for RNA extraction and the levels of the transfected reporter RNA were quantified by RT‐qPCR. FLuc activity was normalized to the renilla luciferase (RLuc) activity to evaluate reporter translation efficiency.

### Study Approval

The Institutional Animal Care and Use Committee of the Dalian Medical University approved the use of animal models in this study (approval no. AEE21015).

All human tumor tissues were obtained with written informed consent from patients or their guardians prior to participation in the study. The Institutional Review Board of the First Affiliated Hospital of Dalian Medical University approved use the tumor specimens in this study (approval no. PJ‐KS‐KY‐2022‐208).

### Statistical Analysis

All data were presented as mean ± SD. Analysis was performed using GraphPad Prism 9.0 software. Two‐tailed student's t test, one‐way ANOVA with Tukey's multiple comparison test or one‐way ANOVA with Dunnett's multiple comparison test were used to evaluate the group difference. The χ^2^ test was used to analyze the association of the expression level of RBMS1 with the patient's clinicopathologic characteristics. Kaplan‐Meier survival analysis and log‐rank test were used to assess survival difference. All statistical tests were considered statistically significant when *P* values less than 0.05.

## Conflict of Interest

The authors declare no conflict of interest.

## Author Contributions

Y.S., D.C., and S.S. contributed equally to this work. Y.W. and W.Z. wrote the manuscript. Y.W. and W.Z. conceived the project and designed the experiments. Y.S., D.C., S.S., M.R., L.Z., H.W., Q.Z., J.Z., G.Z., and Y.Q. designed and performed most of the experiments, whereas C.C., J.Z., H.L., Q.Y., and Q.L. performed data analysis. Y.W. and W.Z. provided funds.

## Supporting information

Supporting Information

Supplemental Table 1

Supplemental Table 2

## Data Availability

The data that support the findings of this study are available in the Supporting Information of this article.
