# Supplementary material for: RBMS1 Coordinates with the m6A Reader YTHDF1 to Promote NSCLC Metastasis through Stimulating S100P Translation
Source: Adv Sci (Weinh). 2024 Feb 11;11(15):2307122. doi: 10.1002/advs.202307122 (PMC11022699; doi:10.1002/advs.202307122)
Supplement: Supplementary file 1 — Supporting Information [file ADVS-11-2307122-s002.pdf]

ADVANCED SCIENCE
Open Access

Supporting Information

for *Adv. Sci.*, DOI 10.1002/advs.202307122

RBMS1 Coordinates with the m^6A Reader YTHDF1 to Promote NSCLC Metastasis through Stimulating S100P Translation

Yu Sun, Dan Chen, Siwen Sun, Menglin Ren, Liang Zhou, Chaoqun Chen, Jinyao Zhao, Huanhuan Wei, Qingzhi Zhao, Yangfan Qi, Jinrui Zhang, Ge Zhang, Han Liu, Qingkai Yang, Quentin Liu, Yang Wang and Wenjing Zhang**

Supporting Information

RBMS1 Coordinates with the m^{6}A reader YTHDF1 to Promote NSCLC Metastasis through Stimulating S100P Translation

Yu Sun#, Dan Chen#, Siwen Sun#, Menglin Ren, Liang Zhou, Chaoqun Chen, Jinyao Zhao, Huanhuan Wei, Qingzhi Zhao, Yangfan Qi, Jinrui Zhang, Ge Zhang, Han Liu, Qingkai Yang, Quentin Liu, Yang Wang and Wenjing Zhang**

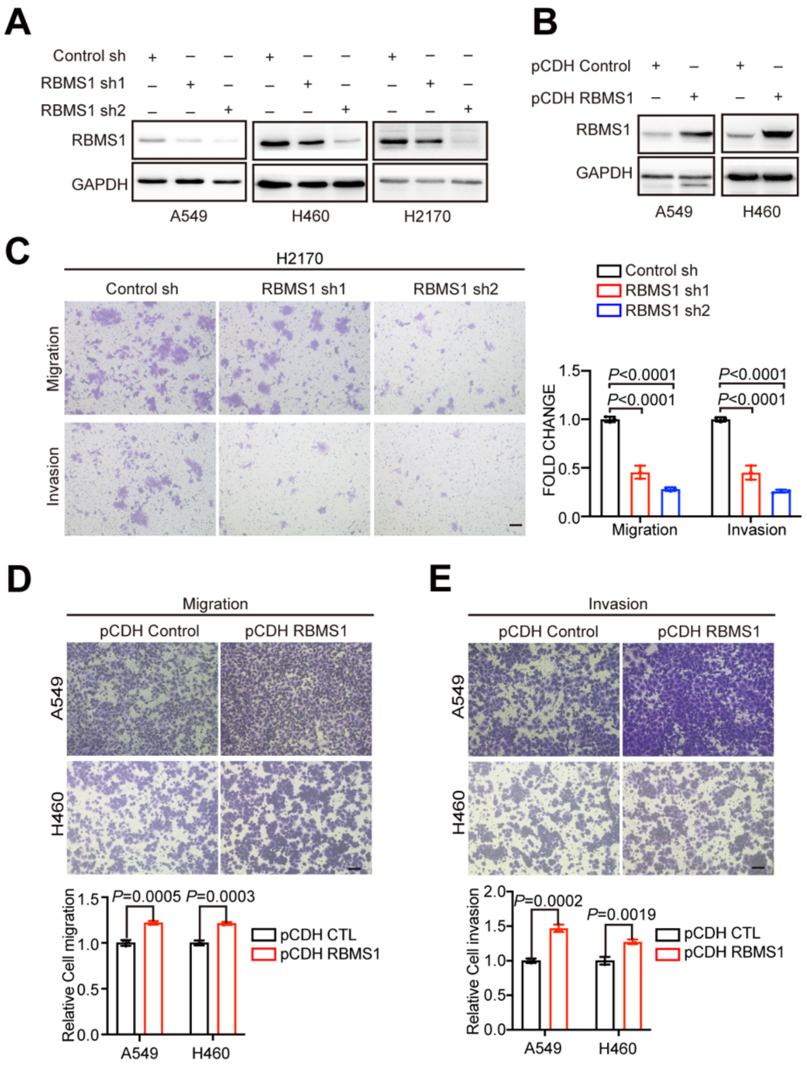

Figure S1. The effect of RBMS1 expression on cell migration and invasion of A549 and H460 lung cancer cells. (**A, B**) A western blot assay was applied to examine the protein levels of RBMS1 in A549, H460 and H2170 cells with stable depletion or overexpression of RBMS1. (C) Effect of RBMS1 knockdown on migration and invasion of H2170 cells evaluated by transwell assays. Scale bars: 100 µm. *P* values were determined using one-way ANOVA with Dunnett's multiple comparison test (n = 3). (**D, E**) Effect of RBMS1 overexpression on migration and invasion of A549 and H460 cells evaluated by transwell assays. Scale bars: 100 µm. *P* values were determined by unpaired Student's *t* test (n = 3).

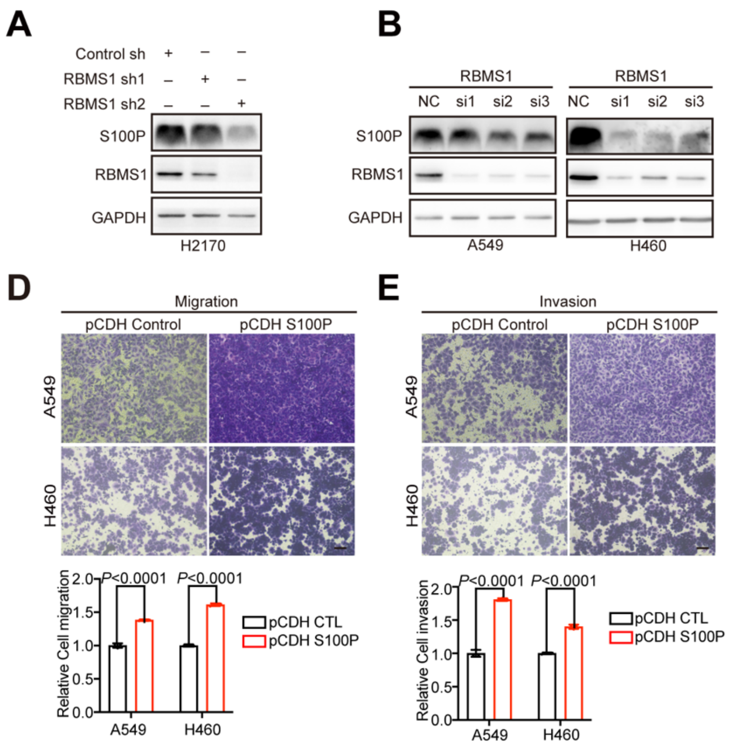

Figure S2. Depletion of RBMS1 inhibits lung cancer metastasis partially by repressing S100P. (**A**) The metastatic protein S100P was examined in RBMS1 knockdown stable H2170 cells. (**B**) The protein level of S100P and RBMS1 were examined in RBMS1 transiently depleted A549 and H460 cells. (**C**) The protein level of S100P were examined in S100P overexpressing A549 and H460 cells. (**D, E**) Effect of S100P overexpression on migration and invasion of A549 and H460 cells evaluated by transwell assays. Scale bars: 100 μm. *P* values were determined by unpaired Student's *t* test (n = 3). (**F**) The protein levels of S100P and RBMS1 were examined with a western blot assay in RBMS1 stably depleted A549 and H460 cells with or without re-expression of S100P.

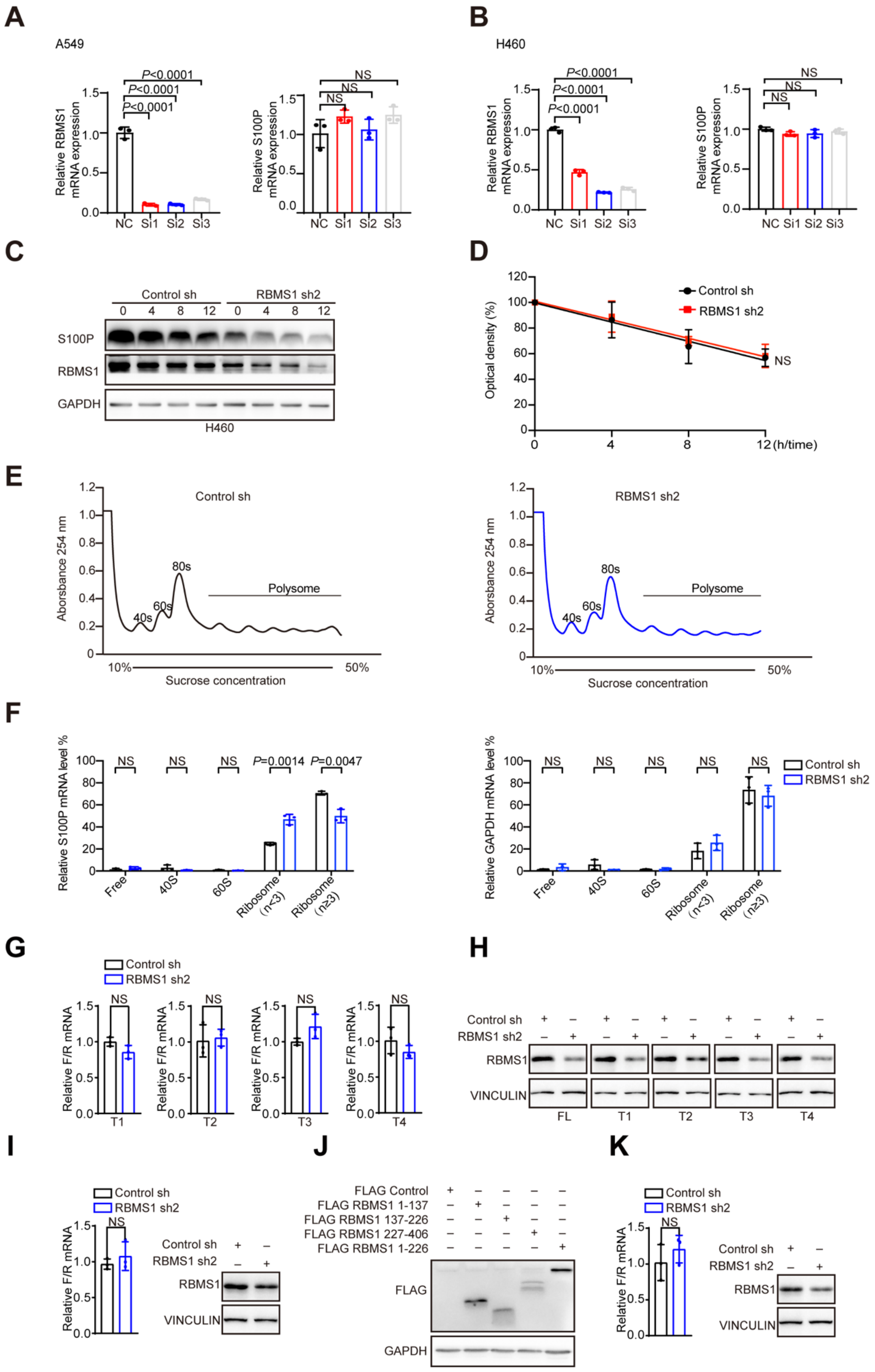

Figure S3. RBMS1 affects translation of S100P. (**A, B**) The mRNA level of S100P

and RBMS1 in RBMS1 transiently knocked down A549 and H460 cells were examined using RT-qPCR. *P* values from ordinary one-way ANOVA with Dunnett's multiple comparison test (n = 3). (**C, D**) RBMS1 stably depleted H460 cells were treated with 100 μg/mL cycloheximide (CHX) at the indicated time points. The protein level of S100P and RBMS1 were measured using a western blot assay. The intensity of S100P was quantified and plotted. Three experiments were conducted with mean ± SD presented. *P* values were determined by two-way repeated measures ANOVA (n = 3). (**E**) Polysome profiling of RBMS1 depleted H460 and control H460 cells were analyzed. (**F**) Analysis of S100P and GAPDH mRNA in the polysomes of RBMS1 depleted H460 cells compared with control H460 cells. n, number of 80S ribosomes. Three experiments were carried out. *P* values were determined by unpaired Student's t test. (**G**) H460 cells were co-transfected with S100P-fluc-T1, S100P-fluc-T2, S100P-fluc-T3 or S100P-fluc-T4, and renilla (loading control), followed by the indicated virus infection of control sh and RBMS1 sh2. The mRNA level of S100P-fluc was examined using RT-qPCR. *P* values were determined by unpaired Student's t test (n = 3). (**H**) H460 cells were co-transfected with S100P-fluc-FL, S100P-fluc-T1, S100P-fluc-T2, S100P-fluc-T3 or S100P-fluc-T4, and renilla (loading control), followed by the indicated virus infection of control sh and RBMS1 sh2. The protein level of RBMS1 was examined using a western blot assay. (**I**) H460 cells were co-transfected with S100P-fluc-FL Mut and renilla (loading control), followed by the indicated virus infection of control sh and RBMS1 sh2. The mRNA level of S100P-fluc was examined using RT-qPCR. P values were determined by unpaired Student's t test (n = 3). The

protein level of RBMS1 was examined using a western blot assay. (**J**) The protein expression levels of Flag-RBMS1 truncations (1-137 aa, 137-226 aa ,227-406 aa and 1-226 aa) in A549 cells were examined using a western blot assay. (**K**) H460 cells were co-transfected with S100P-fluc-FL m^6A Mut and renilla (loading control), followed by the indicated virus infection of control sh and RBMS1 sh2. The mRNA level of S100P-fluc was examined using RT-qPCR. *P* values were determined by unpaired Student's *t* test (n = 3). The protein level of RBMS1 was examined using a western blot assay.

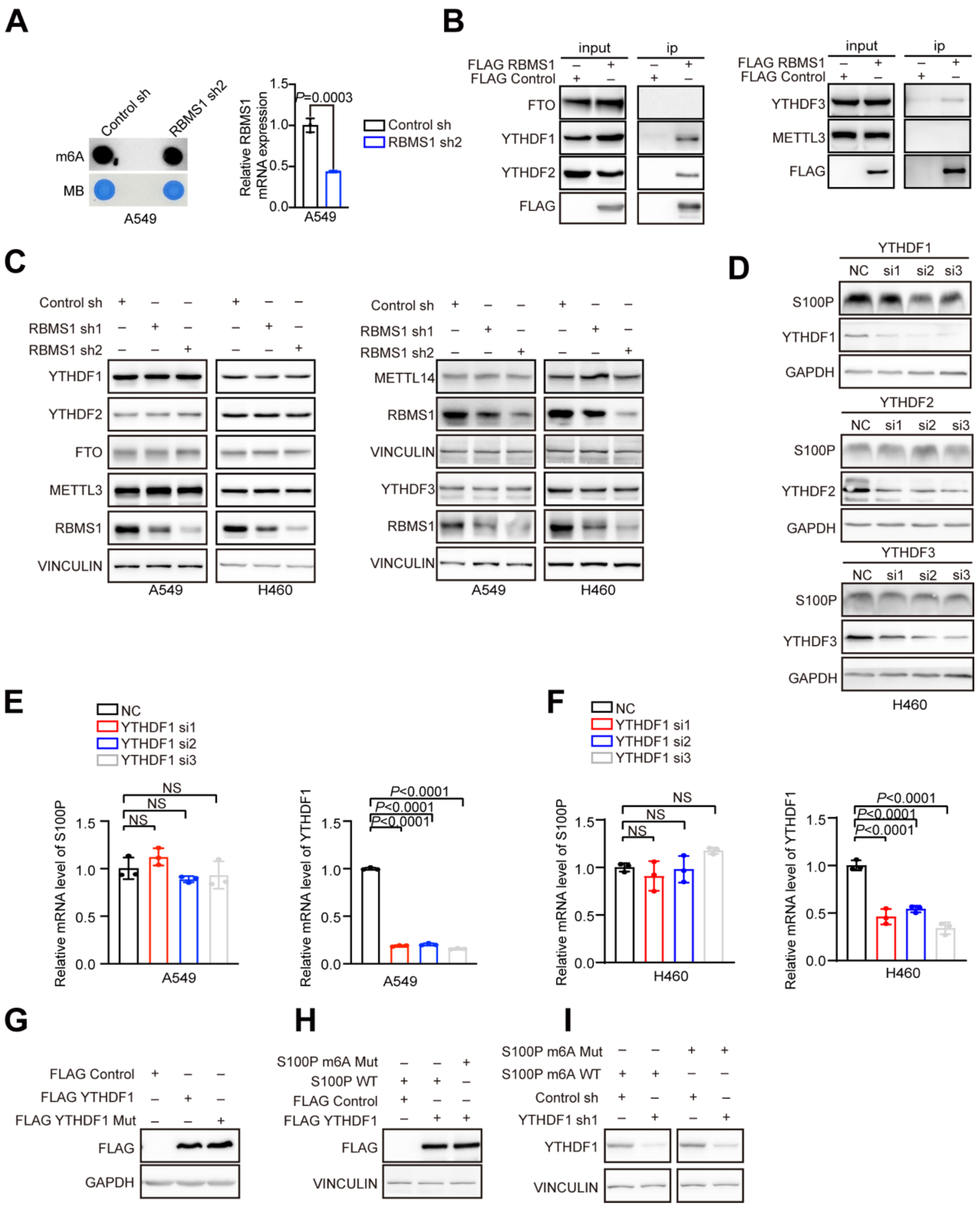

Figure S4. RBMS1 coordinates with YTHDF1 to regulate S100P translation. (**A**) The effects of RBMS1 knock down on global m6A modification in A549 cells. MB: Methylene blue. The mRNA levels of RBMS1 in RBMS1 stable knocked down A549 cells were examined using RT-qPCR. *P* values were determined by unpaired Student's t test (n = 3). (**B**) Immunoprecipitation was performed in 293T cells expressing Flag-RBMS1. The m6A relative proteins were examined using a western blot assay. (**C**) The

m6A relative proteins were examined in RBMS1 knockdown stable A549 and H460 cells. (**D**) The protein levels of S100P were examined in YTHDF1, YTHDF2 and YTHDF3 transiently depleted H460 cells. (**E, F**) The mRNA levels of S100P and YTHDF1 in YTHDF1 transiently knocked down A549 and H460 cells were examined using RT-qPCR. *P* values from ordinary one-way ANOVA with Dunnett's multiple comparison test (n = 3). (**G**) The protein levels of Flag-YTHDF1 wt and Flag-YTHDF1 Mut were measured using a western blot assay. (**H**) pGL3-S100P-WT and PGL3-S100P-m6A mut were transiently into Flag-YTHDF1 stable overexpressing 293T cells. The protein level of Flag-YTHDF1 was examined using a western blot assay. (**I**) H460 cells were co-transfected with S100P-fluc-FL-m6A Mut and renilla (loading control), followed by the indicated virus infection of control sh and YTHDF1 sh1. The protein level of YTHDF1 was examined using a western blot assay.

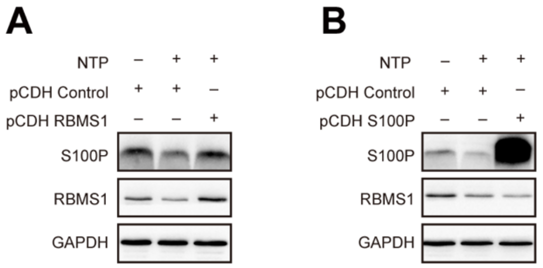

Figure S5. RBMS1 small-molecule inhibitor Nortriptyline hydrochloride (NTP) attenuates tumor metastasis by inhibiting S100P expression. (**A**) The protein levels of S100P and RBMS1 were examined in RBMS1 overexpressed A549 cells or control A549 cells treated with 20 μM NTP. (**B**) The protein levels of S100P and RBMS1 were examined in S100P overexpressed A549 cells or control A549 cells treated with 20 μM NTP.
